# Tsetse fly ecology and risk of transmission of African trypanosomes related to a protected forest area at a military base in the city of Abidjan, Côte d’Ivoire[Fn FN1]

**DOI:** 10.1051/parasite/2023037

**Published:** 2023-09-20

**Authors:** Yao Jean Rodrigue Konan, Djakaridja Berté, Bi Tra Dieudonné Ta, Jean-Paul Demoncheaux, Sylvie Sauzet, Stéphanie Watier-Grillot, Koffi Alain De Marie Kouadio, Louis N’dri, Bamoro Coulibaly, Philippe Solano, Sophie Ravel, Adeline Ségard, Dramane Kaba, Thierry De Meeûs, Vincent Djohan, Vincent Jamonneau

**Affiliations:** 1 Institut Pierre Richet, Institut National de Santé Publique 01 BP 1500 Bouaké Côte d’Ivoire; 2 Université Félix Houphouët-Boigny 01 BPV 34 Abidjan Côte d’Ivoire; 3 Direction interarmées du service de santé pour l’Afrique Centrale et de l’Ouest BP 175 Abidjan Côte d’Ivoire; 4 Université Montpellier, IRD, Cirad, Intertryp 34398 Montpellier France; 5 IRD, Cirad, Intertryp, UMR177 34398 Montpellier France

**Keywords:** *Glossina palpalis palpalis*, *Trypanosoma*, Ecology, Vector control, Abidjan, Côte d’Ivoire

## Abstract

African trypanosomoses, whose pathogens are transmitted by tsetse flies, are a threat to animal and human health. Tsetse flies observed at the military base of the French Forces in Côte d’Ivoire (FFCI base) were probably involved in the infection and death of military working dogs. Entomological and parasitological surveys were carried out during the rainy and dry seasons using “Vavoua” traps to identify tsetse fly species, their distribution, favorable biotopes and food sources, as well as the trypanosomes they harbor. A total of 1185 *Glossina palpalis palpalis* tsetse flies were caught, corresponding to a high average apparent density of 2.26 tsetse/trap/day. The results showed a heterogeneous distribution of tsetse at the FFCI base, linked to more or less favorable biotopes. No significant variation in tsetse densities was observed according to the season. The overall trypanosomes infection rate according to microscopic observation was 13.5%. Polymerase chain reaction (PCR) analyses confirmed the presence of *Trypanosoma vivax* and *T. congolense* forest type, responsible for African animal trypanosomosis. Our findings suggest that there is a risk of introduction and transmission of *T. brucei gambiense*, responsible for human African trypanosomiasis, on the study site. This risk of transmission of African trypanosomes concerns not only the FFCI base, but also inhabited peripheral areas. Our study confirmed the need for vector control adapted to the eco-epidemiological context of the FFCI base.

## Abstract (fr)

Les trypanosomoses africaines, dont les agents pathogènes sont transmis par les mouches tsé-tsé, constituent une contrainte pour la santé animale et humaine. Des mouches tsé-tsé observées dans la base militaire des Forces françaises en Côte d’Ivoire (base FFCI) ont probablement été impliquées dans l’infection et la mort de chiens militaires. Des enquêtes entomologiques et parasitologiques ont été menées pendant la saison pluvieuse et la saison sèche à l’aide de pièges “Vavoua” afin d’identifier les espèces de mouches tsé-tsé, leur distribution, les biotopes favorables et leur source de nourriture ainsi que les trypanosomes qu’elles hébergent. Au total 1185 mouches tsé-tsé de l’espèce *Glossina palpalis palpalis* ont été capturées, ce qui correspond à une densité apparente moyenne élevée de 2,26 tsé-tsé/piège/jour. Les résultats ont montré une distribution hétérogène des tsé-tsé dans la base FFCI en lien avec des biotopes plus ou moins favorables. Aucune variation significative des densités de tsé-tsé n’a été observée en fonction de la saison. Le taux d’infection global par les trypanosomes était de 13,5 % selon l’observation microscopique. Les analyses PCR ont confirmé la présence de *Trypanosoma vivax* et *T. congolense* type forêt, responsable de la trypanosomose animale africaine. Nos résultats suggèrent qu’il existe un risque potentiel d’introduction et de transmission de *T. brucei gambiense* responsable de la trypanosomiase humaine africaine dans la zone d’étude. Ce risque de transmission des trypanosomes africains concerne non seulement l’intérieur de la base FFCI, mais aussi les espaces périphériques habités. Notre étude a confirmé la nécessité de mener une lutte antivectorielle adaptée au contexte éco-épidémiologique de la base FFCI.

## Introduction

Tsetse flies are bloodsucking insects that are found exclusively on the African continent between the 15th degree of north latitude and the 29th degree of south latitude [27]. These flies transmit trypanosomes, which are responsible for human African trypanosomiasis (HAT) caused by *Trypanosoma brucei gambiense* in West and Central Africa and *T. b. rhodesiense* in East Africa [7]. They also transmit trypanosomes that cause animal African trypanosomosis (AAT), mainly *T. congolense*, *T. vivax*, and *T. b. brucei* [15, 17]. Significant efforts to control HAT since the 1990s have greatly reduced the prevalence of the disease. The World Health Organization (WHO) now aims to interrupt the transmission of HAT by 2030 [22]. AAT, due to its impact on agriculture and livestock, is one of the main obstacles to the economic development of affected rural areas on the African continent [4]. In 2002, the African Union established the Pan-African Campaign for the Eradication of Tsetse and Trypanosomiasis (PATTEC) with the goal of eradicating it from the African continent [31].

In Côte d’Ivoire, nine species and subspecies of tsetse flies have been identified [40], including *Glossina palpalis palpalis*, the main vector of HAT and AAT in West Africa [13]. In December 2020, the WHO validated the elimination of HAT as a public health problem in this country [30, 48]. However, a few cases are still reported in the most recent endemic foci [35, 36]. AAT continues to persist across Côte d’Ivoire, and although the current situation is not well-documented, high prevalence have been recently observed in the northern regions [2, 6] and the central-western part of the country [44, 60].

The military base of the French Forces in Côte d’Ivoire (FFCI base), located in Abidjan, provides a favorable biotope for tsetse flies due to the shade and humidity provided by its vegetation. The presence of wild and domestic animals also serves as a source of blood meals for the flies. Cases of AAT caused by forest type *T. congolense* were identified in the late 1990s and early 2000s in military working dogs, and the presence of *G. p. palpalis* was documented [34, 37, 61]. These cases of canine trypanosomosis, along with the presence of tsetse flies, indicated not only a risk of trypanosome transmission to animals residing within the FFCI base but also to domestic animals circulating around the base. Furthermore, the risk of introducing and transmitting *T. b. gambiense* within or outside the base could not be ruled out for individuals who had stayed in endemic areas in the central west part of the country or in another HAT focus in Africa. In response to this situation, vector control (VC) measures were implemented in 2005 and 2006 to reduce tsetse density and minimize the risk of trypanosome transmission (DK, personal observation). However, a decade after the end of this VC campaign, a new fatal case of canine AAT was reported at the FFCI base [9], and the presence of tsetse flies was once again reported.

The objective of the present study was to evaluate the entomological and parasitological situation regarding African trypanosomoses in 2020 within the FFCI base. Special attention was paid to the ecology of tsetse flies according to the seasons, aiming to establish an effective and sustainable VC campaign to interrupt trypanosome transmission in this environment.

## Materials and methods

### Study area

**Figure 1:**
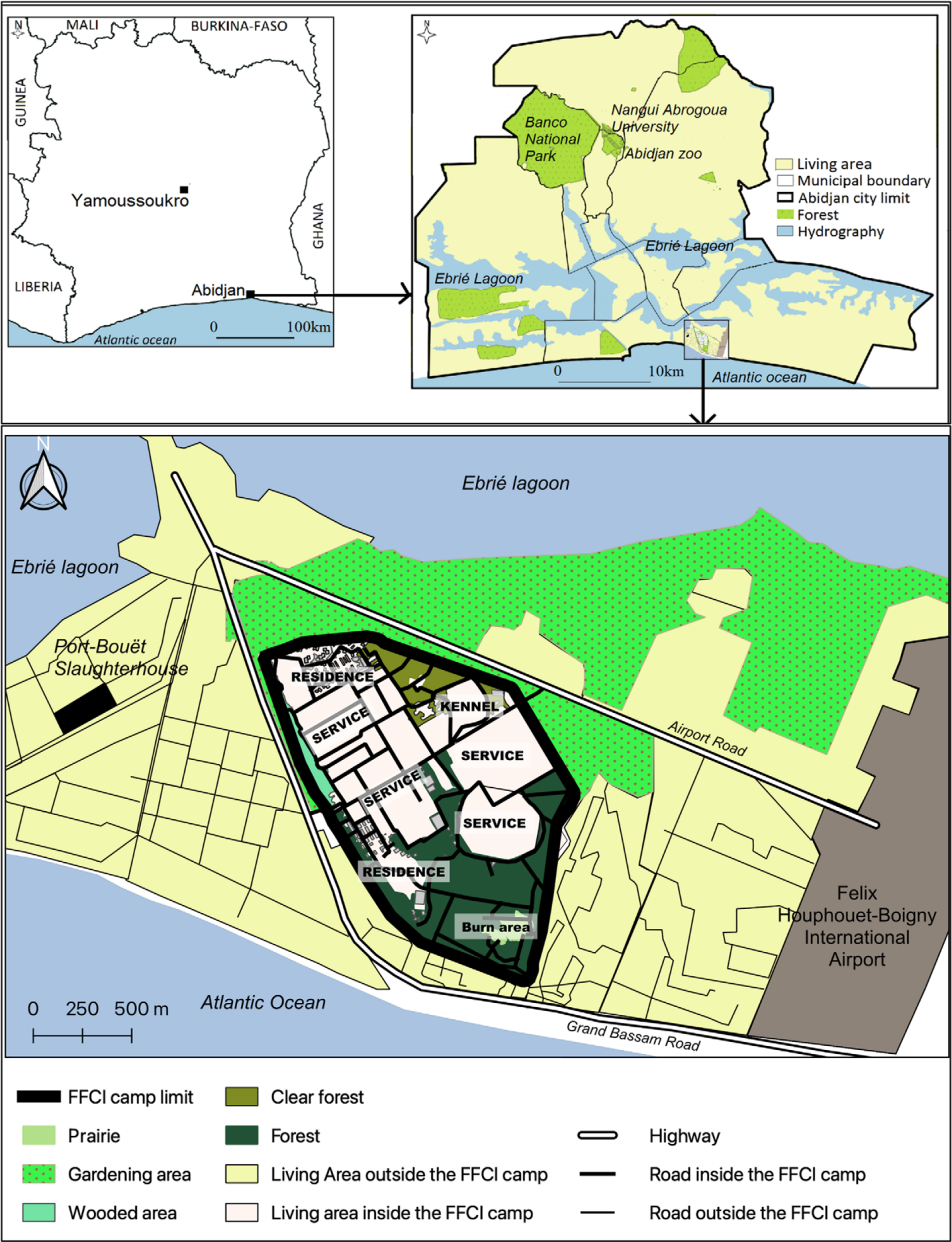
Study area (Source: Institut Pierre Richet, 2023, published with authorization).

This study was conducted within the FFCI base, situated in the commune of Port-Bouët, south of Abidjan (Fig. 1). The base is located between the Atlantic Ocean, the Ebrié lagoon, and Félix Houphouët-Boigny International Airport. The climate is characterized as humid tropical, with four distinct seasons: a long rainy season from April to July, a short rainy season from September to November, a short dry season from August to September, and a long dry season from December to March. Temperatures range from 25 °C in August, considered the coldest month, to 29 °C in April, considered the hottest month. The average annual rainfall is 1847.6 mm. Relative humidity ranges from 82% in January to 92% in August, with an average of 88% [45]. The FFCI base covers an area of 1.7 km^2^, with a dense evergreen humid forest (about 0.5 km^2^) in its southern part. Several species of wild animals, including bushbuck, civets, rodents, squirrels, monitor lizards and various snake species, are regularly observed in this area. Within the base, there is a kennel housing “military working dogs” and other domestic animals such as “civilian dogs,” cats and goats are also present. Abidjan’s largest livestock market and the main slaughterhouse are located a few hundred meters from the base. The area south of the base is entirely inhabited, while the area north serves as a market gardening area (Fig. 1).

**Figure 2:**
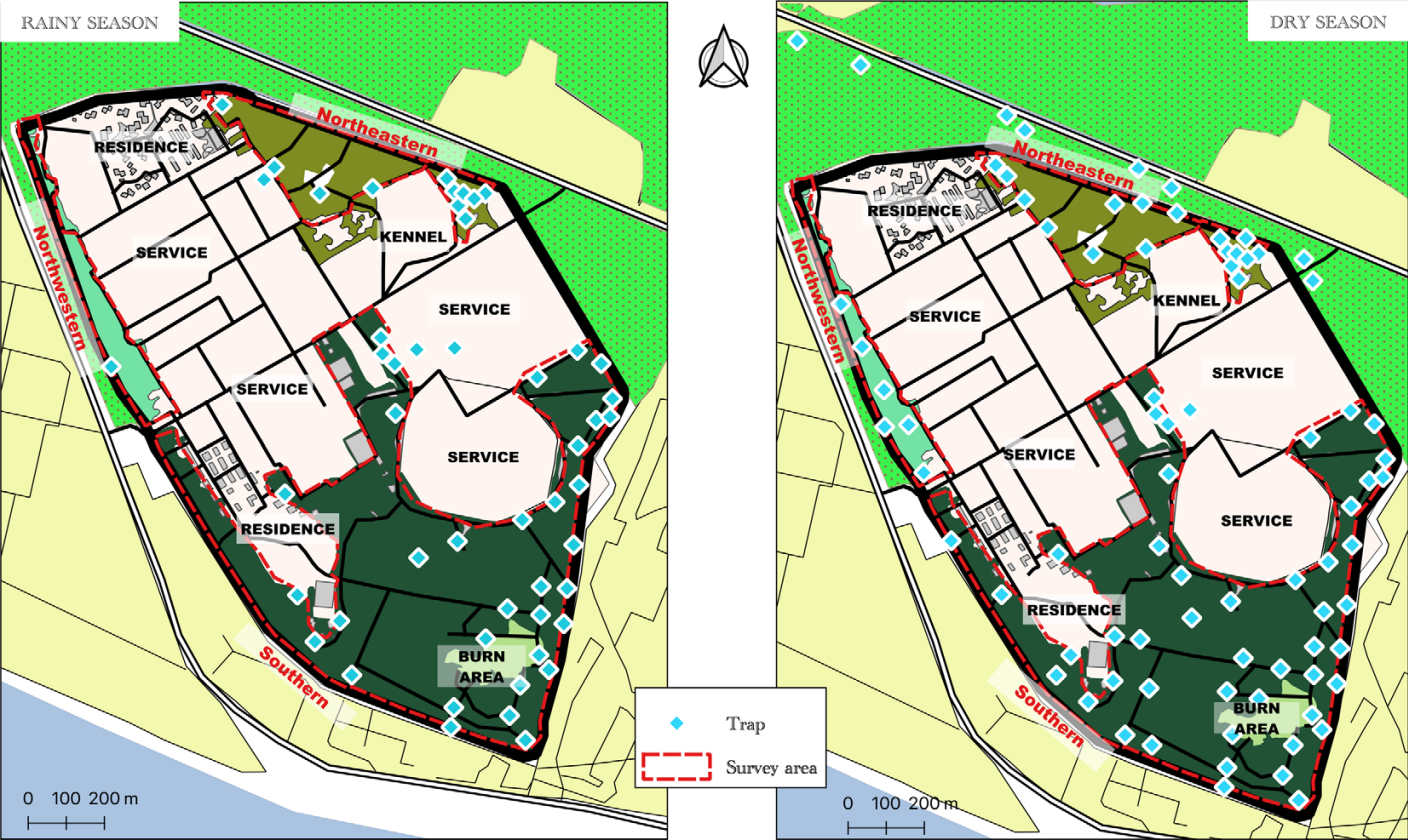
Distribution of catch traps set in May 2019 (left) and January 2020 (right) (Source: Institut Pierre Richet, 2023, published with authorization).

The FFCI base is divided into an inhabited area consisting of residential, service, and kennel areas, and three zones with vegetation that could potentially be favorable for tsetse flies: (i) the Southern Zone of the base, characterized by dense evergreen forest surrounding a meadow where green waste is burned (called the burn area); (ii) the Northeastern Zone, located near the kennel, with a clear forest area with open undergrowth; and (iii) the Northwestern Zone, which is wooded but lacks undergrowth vegetation, situated between the enclosure and the inhabited areas (Figs. 1 and 2).

### Entomological surveys

Two entomological surveys were conducted to assess the epidemiological situation within the FFCI base. The first survey took place during the long rainy season (May 2019), where 50 monoconical traps (Vavoua) [39] were placed in locations favorable for tsetse host contact within the base (Fig. 2). During the second survey in the long dry season (January 2020), a total of 72 traps were deployed. This included 48 traps at similar locations as in May 2019 (with two locations inaccessible in January 2020), and an additional 24 traps at new locations. Furthermore, nine additional traps were set up in the market gardening area to the north, outside the base (Fig. 2). All traps were georeferenced using a GPS (Garmin 64^®^). The traps were left in place for four consecutive days, and tsetse flies were collected every 24 h (four harvests in total). Captured tsetse flies were identified by species and sex using a binocular magnifying glass and an identification key [49].

### Detection and identification of trypanosomes

For all traps, 20% of the captured tsetse flies were dissected under a binocular magnifying glass [62]. The proboscis, midgut, and salivary glands were isolated for trypanosome detection using an optical microscope (X400). If trypanosomes were observed in at least one of the three organs, the organs were individually collected in Eppendorf^®^ tubes containing 25 μL of distilled water and stored at −20 °C for subsequent trypanosome species identification using polymerase chain reaction (PCR) [32, 59].

DNA extraction was performed using two different protocols: (i) 5% Chelex 100^®^ (a chelating ion exchange resin, Bio-Rad, Hercules, CA, USA) for samples collected in May 2019; (ii) DNeasy^®^ (QIAGEN, Hilden, Germany) blood and tissue extraction kits for samples collected in January 2020, following the manufacturer’s instructions. Different primers were used depending on the sample collection date. PCR analysis of samples collected in May 2019 was conducted using TRYP1S/1R primers to diagnose *T. brucei* s.l., *T. vivax*, *T. congolense* forest type, and *T. congolense* savannah type [16]. For samples collected in January 2020, additional PCRs were performed to enhance sensitivity and specificity for detecting *T. brucei* s.l. using 18S-F/R primers [14], and Tg19-F/R primers for *T. brucei gambiense* [50].

### Blood meal analysis

During the dissection of tsetse flies, any presence of undigested blood meals was collected on Whatman FTA^®^ paper number 4. The origin of this blood was determined by PCR targeting the cytochrome B gene, a phylogenetic marker in vertebrates [20]. PCR amplification was carried out using primers designed to amplify a 358 base pairs (bp) region of the cytochrome B gene in vertebrate mitochondrial DNA. The PCR amplicons were purified using a QIAquick PCR^®^ purification kit (QIAGEN, Valencia, CA, USA) for bidirectional sequencing. The obtained DNA sequences were aligned with the GenBank sequence base using the BLAST (Basic Local Alignment Search Tool) method to identify the origin of the blood.

### Data analysis

Statistical tests were performed using R version 4.0.2 [28]. The number of tsetse flies was expressed as the apparent density per trap per day (ADT), which represents the average number of tsetse flies captured per trap per day. A log-normal regression (Poisson) was used to assess the variation in ADT of tsetse flies according to the season, zone and their interaction, i.e., ADT ~ Season + Zone + Season:Zone. Significance of each term was tested with chi-square tests of model comparisons with the Benjamini and Hochberg (BH) correction of *p* values [8] (script 1, Supplementary material 1).

The sex-ratio (SR) was calculated as the number of males divided by the number of females. The significance of deviation from the expected proportion of females (50%) was examined for each season/zone combination using exact binomial tests, followed by a BH correction (script 2, Supplementary material 1).

Logistic regression was employed to investigate the influence of season and sex on the presence or absence of trypanosomes in tsetse flies (determined by microscopic examination). The optimal logistic model selected via the stepwise process was Infected ~ Gender + Season + Zone + Gender:Season + Gender:Zone + Season:Zone + Gender:Season:Zone. The significance of the different parameters was then assessed using chi-square tests for model comparisons with the BH correction (script 3, Supplementary material 1).

## Results

### Entomological parameters

**Figure 3:**
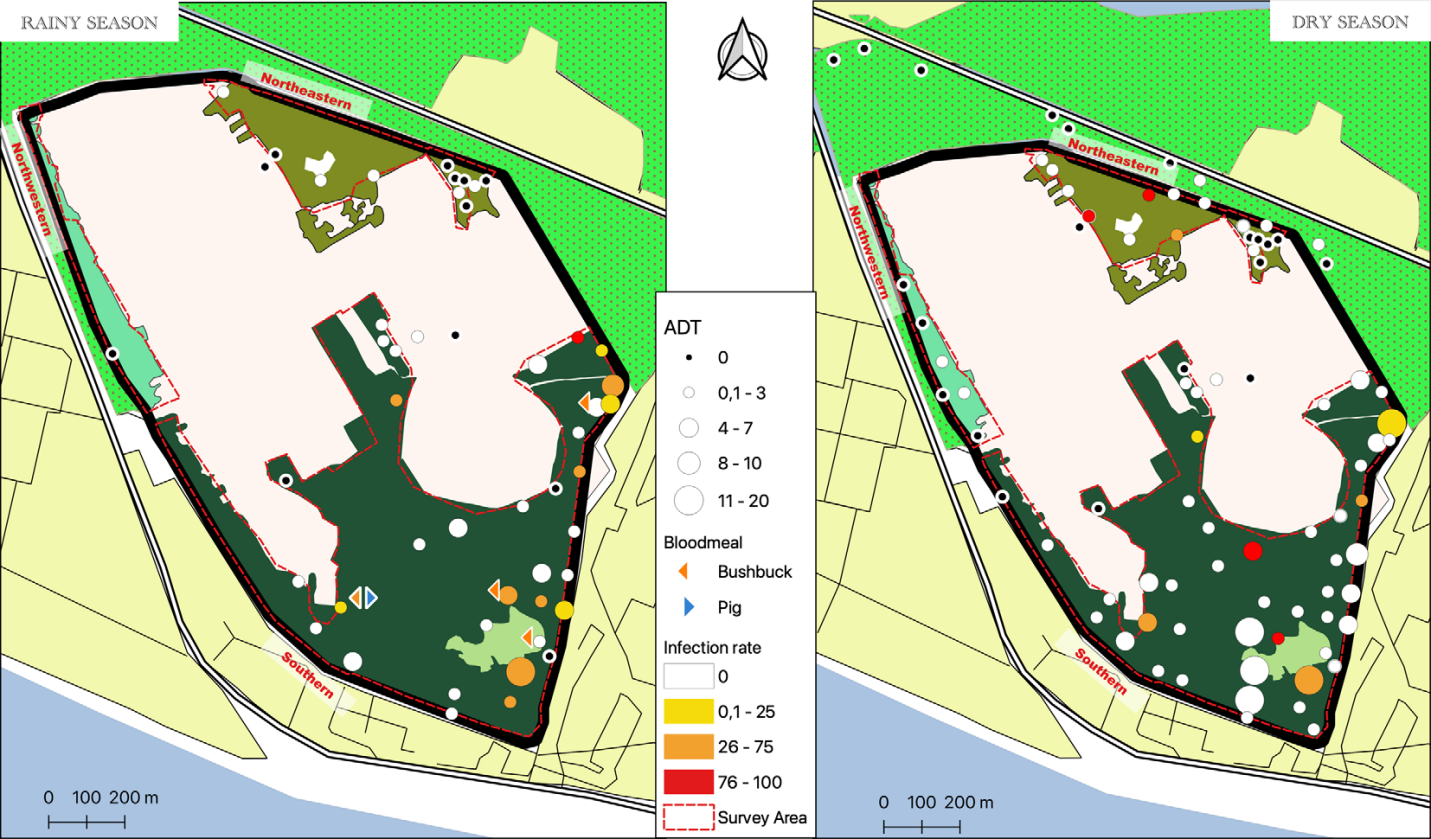
Distribution of ADTs, infection rates and blood meals of tsetse flies in the FFCI base according to season (Source: Institut Pierre Richet, 2023, published with authorization).

**Figure 4:**
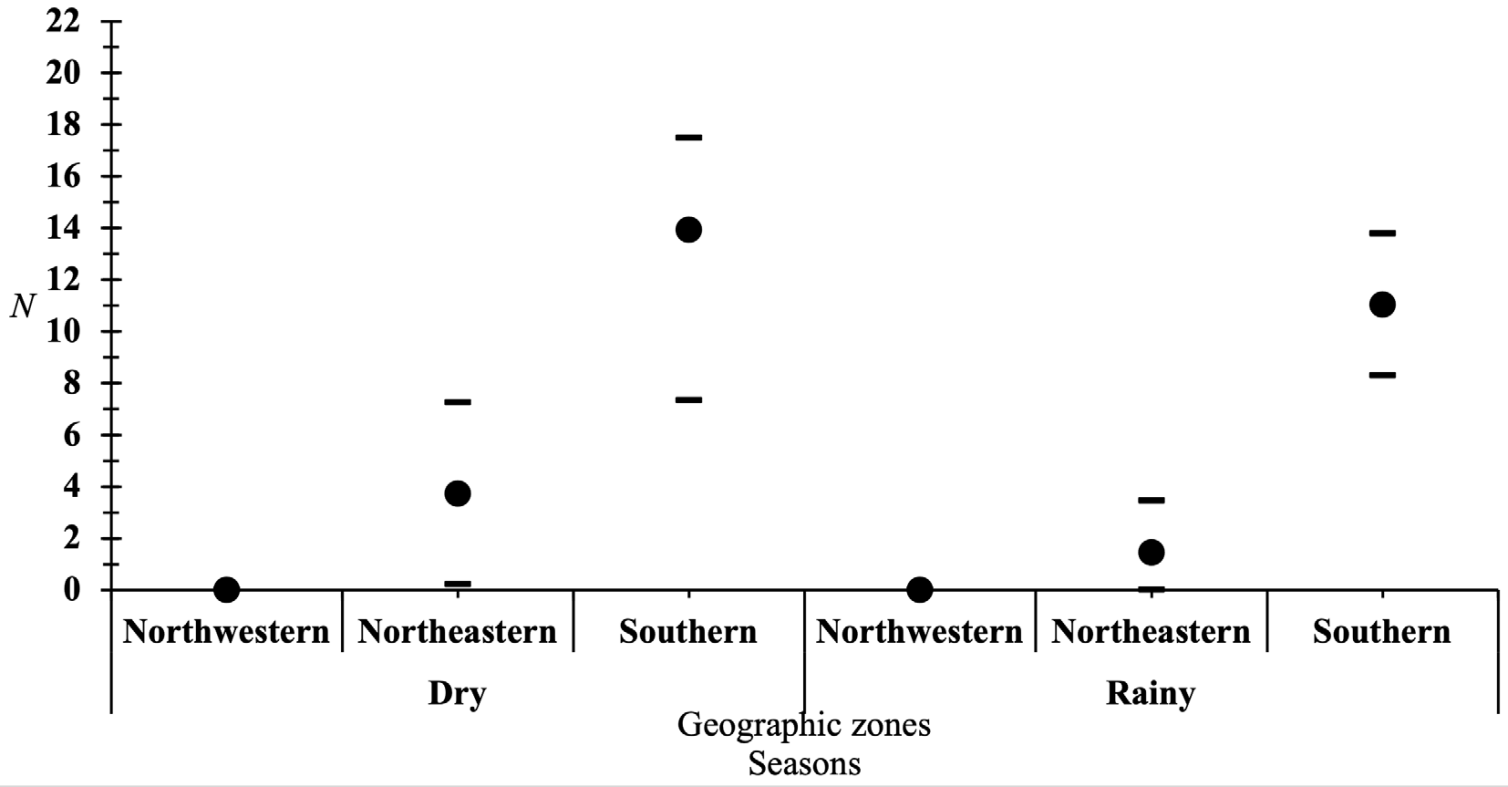
Total number of tsetse flies caught (*N*) in the different areas of the FFCI base and the different seasons. 95% confidence interval $\mathrm{CI}=N\pm t_{\alpha =0.05,\;\nu \;=n-1\;}\times \;(s/\sqrt{n}$), where CI is the confidence interval; *N* is the sample mean; *t* is the critical value of the Student distribution corresponding to the confidence level chosen and the degrees of freedom associated with the sample; *n* − 1 is the degrees of freedom, where n is the sample size; *s* is the standard deviation of the sample; $\sqrt{n}$ is the square root of the sample size.

A total of 1185 tsetse flies were captured (Supplementary material 2), all of which belonged to *G. p. palpalis*. Among them, 1182 were captured inside the FFCI base, and three were found in the market gardening area north of the FFCI base (Fig. 3). The ADT was 2.26 tsetse flies, with a spatial distribution that varied from 0 to 19.5 across all captures. The highest ADTs were observed in the Southern Zone, particularly around the burn area. Tsetse flies were also captured along the perimeter and around the inhabited areas of the base (Fig. 3). The log-normal regression (script 1, Supplementary material 1) revealed: (i) a highly significant influence of the zones (*p* = 6.600e−16) and the season (*p* = 6.219e−08) (Fig. 4). Given the sample size, an extremely low *p* value of 10e−16 was not expected, which may indicate a lack of applicability of the chi-square test. To validate the consistency of the obtained *p* values, we reanalyzed the data using a two-factor ANOVA with the R software, and it showed that only the areas had a significant effect (*p* = 0.00026) (script 1, Supplementary material 1).

Out of the 1185 captured tsetse flies, 632 were males and 553 were females, resulting in a male biased sex ratio (SR) of 1.14. There was a significant difference (*p* = 0.023) in the number of males and females captured, favoring males. Considering the area and the season, the exact binomial test (script 2, Supplementary material 1) revealed that this significant difference only occurred in the Southern Zone during the dry season (*p* = 0.0008). In the Southern Zone during the rainy season and in other areas regardless of the season, the SRs were balanced (*p* ≥ 0.6).

### Parasitological investigation

Microscopic detection of trypanosomes was performed on 232 dissected tsetse flies (101 males and 131 females) of which 31 (5 males and 26 females) were found to be infected (13.5%) (Supplementary material 2). The optimal logistic model selected via the stepwise process was Infected ~ Gender + Season + Zone + Gender:Zone + Season:Zone (script 3, Supplementary material 1). Results of the tests are in Table 1. Analysis performed on tsetse infections indicated that females had a significantly higher infection rate than males (*p* = 0.003). Season, zone and pairwise interactions were retained in the optimal model but were not significantly associated with the infected status. Trypanosomes were observed either in the proboscis only (*n* = 18), midgut only (*n* = 6), or both the proboscis and midgut (*n* = 7). No infections were observed in the salivary glands. While the majority of infections were found in the Southern Zone (29), two infected tsetse flies were also observed around the kennel in the Northwestern Zone during the dry season (Fig. 3).

**Table 1:** Results of the ANOVA tests of individual variables in the optimal logistic regression model of presence/absence of trypanosomes in tsetse flies. *p* values are presented before and after the Benjamini and Hochberg (BH) correction.

Factors	*p* value	*p* value after BH correction
Gender	0.0006	0.003
Season	0.098	0.14
Zone	0.2	0.2
Gender:Zone	0.03	0.08
Season:Zone	0.11	0.14

During the rainy season, 21 out of 148 dissected tsetse flies were found to be infected (14.2%), and during the dry season, 10 out of 84 dissected tsetse flies carried trypanosomes (11.9%). The logistic regression analysis showed no significant difference (*p* = 0.14) in the infection rate when considering the two seasons separately (script 3, Supplementary material 1).

Since no infections were observed in the salivary glands, the corresponding samples were not tested by PCR. PCR tests were conducted on the proboscis and midgut of the 31 tsetse flies found infected through light microscopy, except for the midgut of tsetse No. 88 (captured in the rainy season) which could not be collected (Supplementary material 3).

A total of 16 tsetse flies (51.6%) appeared positive for TRYP1S/1R PCR, including 10 tsetse flies infected with *T. vivax* (32.3%) and 6 tsetse flies infected with *T. congolense* forest type (22.6%). No infections with *T. congolense* savannah type, *T. brucei* s.l., or *T. b. gambiense* were detected. PCR tests were negative for 23 organs of tsetse flies that tested positive through microscopy. The 10 *T. vivax* PCR-identified infections were detected in the proboscis including nine for which trypanosomes were observed by microscopy. *T. congolense* forest type infections were detected by PCR in 3 proboscis (including two for which trypanosomes were observed by microscopy) and 4 midgut samples (including three for which trypanosomes were observed by microscopy) (Supplementary material 4). One tsetse fly (No. 36) was infected in both the proboscis and midgut with *T. congolense* forest type.

### Trophic preference

Five blood meals were collected from tsetse flies dissected exclusively during the rainy season in the southern region, and these samples contained sufficient DNA from the cytochrome B gene for analysis. One blood meal exhibited 99% similarity with the pig sequence (*Sus scrofa domesticus*), while four blood meals showed 99% similarity with the bushbuck sequence (*Tragelaphus scriptus*) (Supplementary material 2 and Fig. 3).

## Discussion

Entomological surveys conducted at the FFCI base in Abidjan have revealed the presence of a single species of tsetse fly, *G. p. palpalis*, which is the main vector for trypanosomes causing HAT and AAT in the southern region of Côte d’Ivoire [24, 40, 46]. These findings confirm the results of previous entomological surveys carried out in the same area in the early 2000s [34, 37]. The same species has also been observed in Banco National Park, Nangui Abrogoua University, and the Zoological Park in the northern part of the city of Abidjan (Fig. 1) [1, 3]. Unlike tsetse flies belonging to the *morsitans* and *fusca* groups, which tend to decline due to the decrease in wildlife and habitat loss [26, 53, 54], those from the *palpalis* group are more resilient and can adapt to peri-domestic environments and even large urban centers in certain favorable locations [1, 3, 5, 12, 19, 46, 47, 55]. This adaptability can promote contact between hosts and vectors, leading to the transmission of HAT, as observed in the foci of Daloa in the late 1990s [21] and Sinfra in Côte d’Ivoire [38]. This resilience may be attributed to its food eclecticism [11, 27].

The ADTs observed at the FFCI base, comparable to those observed in the early 2000s [34, 37], are high compared to those observed during recent entomological surveys conducted at other study sites in Côte d’Ivoire [6, 18, 29]. This confirms that the FFCI base has biotopes that are particularly favorable to tsetse flies, with the presence of more or less dense forest areas and a variety of wild and domestic animals, as well as humans, which serve as potential food sources. Additionally, we observed sandy soil throughout the base, which provides an ideal substrate for larviposition and enables larvae to easily burrow into the soil, facilitating their development into the adult stage under optimal conditions.

The distribution of tsetse flies in the different areas surveyed was not uniform, as had already been observed during the entomological surveys conducted in 2003 and 2004 [34]. In our study, tsetse flies were more abundant in the Southern Zone, particularly around the burn area. This could be explained by the presence of a particularly favorable biotope with dense forest relic that offer optimal conditions of humidity, shade, and temperature. Additionally, the constant presence of protected wild animals, such as bushbuck (*Tragelaphus scriptus*), which have been described as preferred hosts of *G. p. palpalis* [10, 11, 40, 57], and which we were able to observe in this area, contributes to the availability of food sources for tsetse flies. The analysis of blood meals collected during this study confirmed this observation and the trophic preference of tsetse flies for this animal. Other mammal species (such as civets, *Civettictis civetta*) and reptiles (turtles, snakes, monitor lizards) observed in the area also serve as food sources for tsetse flies.

The ADTs were lower in the Northwestern Zone of the FFCI base. This is probably due to the more open forest, which is less conducive to tsetse flies. Although we did not observe any blood meal taken from the dog, it is likely that the presence of the kennel plays an attractive role for tsetse flies, which can feed on this animal [11]. The ADTs were very low in the Northeastern Zone of the base, an area devoid of dense vegetation and wildlife due to its high degree of urbanization. Although no blood meals taken from humans were detected during our study, it is likely that some tsetse flies, particularly those caught on the outskirts and even in residential areas, occasionally feed on humans.

It should be noted that a small number of tsetse flies were caught outside the base, in the market gardening area, which is not a suitable habitat for tsetse due to the nature of the vegetation and the significant use of inputs, including insecticides. This observation demonstrates that although tsetse flies find a favorable habitat within the FFCI base, they may still venture outside the base either to disperse or to feed on available hosts. The identification of a blood meal taken from a pig, an animal not reported at the base, confirms this observation. We have observed that domestic animals, including pigs, are raised in inhabited areas on the outskirts of the base. Additionally, livestock (such as cattle, sheep, and goats) pass through the market gardening area, due to the presence of the livestock market and slaughterhouse near the base (Fig. 1). Although we have not explicitly emphasized it, it is possible that some tsetse flies feed on these domestic animals, which can come from various locations in Côte d’Ivoire, or even Burkina Faso and Mali. On the other hand, it is unlikely that tsetse flies from the FFCI base can reach the slaughterhouse to take their blood meal. Firstly, they have a food source available on-site, and secondly, the journey (approximately 1 km for the shortest distance) is lengthy for a tsetse fly through such a highly urbanized area (Fig. 1).

We did not observe significant seasonal variation in ADT. These findings differ from those of other studies where lower ADTs were observed during the dry season, which affects vegetation and makes habitats less suitable for tsetse flies [3, 40]. The vegetation within the FFCI base, in general, is important and well-preserved in certain areas, exhibiting strong resistance to the dry season. This provides optimal living conditions for tsetse flies throughout the year. In the rainy season, heavy rainfall can reduce trap visibility and attractiveness while also limiting the activity of tsetse flies.

The observed infection rate in this study confirms the occurrence of trypanosome transmission at the FFCI base. Specifically, we identified *T. vivax* and *T. congolense* forest type, two parasites responsible for AAT. *T. congolense* forest type has previously been implicated in the death of military working dogs in 2001, 2002, and 2018 [9, 34, 37, 61]. It is possible that military working dogs became infected at the kennel or nearby training ground, where infected tsetse flies were captured, or during patrols within and around the base. The presence of *T. congolense* forest type and *T. vivax* had previously been observed in dissected tsetse flies during entomological surveys conducted in 2003 and 2004 [34]. The persistence of these two trypanosome species within the base is likely due to the presence of protected wild animals that serve as reservoirs for the parasites [43]. We have specifically demonstrated that tsetse flies feed on bushbuck, which are not only described as one of the preferred hosts of *G. p. palpalis* [10, 11, 40, 57], but also exhibit a high tolerance to infection [41, 42]. Additionally, although less common, it is possible that tsetse flies can become infected by animals present or passing outside the base (as mentioned earlier) and contribute to the transmission of trypanosomes between these two areas.

The infection rate did not show significant variation according to the season. This could be attributed to the small sample size, but it may also indicate ongoing and consistent transmission of trypanosomes throughout the year. This could be facilitated by the continuous presence of hosts, regular sources of food, and reservoirs of parasites.

Neither *T. b. brucei* nor *T. b. gambiense* or *T. congolense* savannah type were detected within the base. However, the possibility of these parasites being introduced by humans and/or domestic animals cannot be ruled out, whether they reside or temporarily stay inside or outside the base. This may be particularly relevant for military personnel who may be exposed during their stays in HAT endemic areas in Africa [23], or domestic pigs suspected of serving as reservoirs for human and animal trypanosomes in Côte d’Ivoire [44, 56, 60]. Therefore, the risk of transmission cannot be excluded within and between these two areas. It is important to note that almost half of tsetse flies for which trypanosomes were observed by microscopy in the proboscis and midgut were not positive in PCR. These infections are likely due to other trypanosome species which can infect tsetse flies as reported elsewhere [58]: examples include reptilian trypanosomes such as *T. grayi*, and ubiquist non-pathogenic trypanosomes such as *T. theileri*.

The SRs in tsetse populations can vary depending on several factors such as capture technique, location, time of capture, species, vegetation, and season. Generally, it is biased towards females due to their longer lifespan [25, 40] and/or the increased appetite during the gestation period, making them more attracted to visually appealing traps like the Vavoua trap [39]. This was observed at the FFCI base during the entomological surveys conducted in 2003 and 2004 [34]. In our study, the SR favored males in the Southern Zone of the base. This could be attributed to the abundance of food sources near their resting areas, which may limit the movement of female tsetse and decrease their likelihood of being attracted to and caught by traps. Males, known to be more active than females in their pursuit of mating opportunities [40], would thus be captured more frequently than females in this area. A similar hypothesis was proposed in a recent study conducted in southern Chad, where it was observed that the SR of *Glossina fuscipes fuscipes* populations captured by biconical traps varied with population density [51]. Other unidentified factors are likely to be involved as well.

Females were significantly more infected than males. This can be explained by the fact that females, in order to ensure the maturation of larvae, take a greater number of blood meals at shorter intervals. This biological characteristic provides them with more opportunities to become infected and also to transmit the parasite multiple times to their hosts [33]. These observations support the existence of a preferential cycle between bushbuck – tsetse flies – bushbuck, with occasional involvement of other hosts such as military working dogs. This cycle contributes to the maintenance of the tsetse population and the transmission of animal trypanosomes at the FFCI base, which has a limited surface area with a preserved forest relic.

This study conducted at the FFCI base thus revealed several key findings: (i) the presence of a population of *G. p. palpalis* with particularly high densities due to favorable biotopes, (ii) the occurrence of active transmission of *T. vivax* and *T. congolense* forest type, which are responsible for the previously reported AAT cases, (iii) a risk of introduction and transmission of *T. b. gambiense*, the causative agent of HAT, and (iv) a potential risk of trypanosome transmission between the inside and outside of the base, posing a potential threat to humans and domestic animals residing there.

These results confirmed the need to implement a vector control strategy considering the ecological, entomological, and parasitological parameters associated with this tsetse fly population. One crucial consideration was to choose a method that minimally affects non-target organisms, especially the bees from the numerous beehives established at the base for honey production. We proposed use of West African Tiny Targets (TTs) impregnated with insecticide [52]. Previous studies have demonstrated their high efficacy in various intervention areas targeting *G. palpalis* [30]. In the proposed strategy, TTs were placed considering our findings, particularly the heterogeneous distribution of ADTs, i.e., in (i) all forested areas of the base, (ii) around all anthropized/inhabited areas, and (iii) outside the base to cover the gardening area neighboring the base limits. The objective was to quickly interrupt trypanosome transmission, reducing tsetse fly densities by at least 90% after the first TT deployment. Sustainable management should be considered, which would entail regular entomological surveys to determine the redeployment strategy in terms of frequency and TT number, and the duration and effectiveness of the intervention. Considering that the tsetse fly population at the FFCI base is likely isolated due to the absence of nearby vegetation formations (Fig. 1), the possibility of total eradication of tsetse flies should be studied.

## Supplementary material

The Supplementary materials of this article are available at https://www.parasite-journal.org/10.1051/parasite/2023037/olm *Supplementary material 1*. Scripts with details of the data analysis.
*Supplementary material 2*. Number of tsetse caught per trap by sex, season, and area.
*Supplementary material 3*. Infections and blood meals on dissected tsetse.
*Supplementary material 4*. Results of PCR analyses on organs of infected tsetse.

### Legends for supplementary material

Sex Ratio: calculated only if number of male and female > 0

B02 and B48: traps not installed in January 2020

ADT: apparent density per trap per day

Ind_flies: Individual tsetse fly identification

Organs: Tsetse organs

PR: proboscis

MI: midgut

*Tv: Trypanosoma vivax*

*Tc*f*: Trypanosoma congolense* forest type

TRYP1S/1R: PCR primers pan trypanosomes

18S-F/R: PCR primers specific to *Trypanosoma brucei* s.l.

Tg19-F/R: PCR primers specific for *Trypanosoma brucei gambiense*

Infection: Infection by trypanosomes

NA: Not available

PR+: Trypanosomes found in the proboscis

MI+: Trypanosomes found in the midgut

PR+/MI+: Trypanosomes found in both the proboscis and midgut

BloodMealAnalysis: Origin of the blood meal analyzed
